# Establishing high risk factors for recurrence in stage I/II colorectal cancer: a metropolitan perspective

**DOI:** 10.1186/s12957-025-03823-0

**Published:** 2025-06-18

**Authors:** Phoebe C. Wood, Andrew P. Zammit, Baillie Ferris, Fraser H. Simpson, Matthew Burge, Robert Franz, Yiu Ming Ho, Harish Iswariah, Peter Yuide, Derek Mao, Manju Chandrasegaram

**Affiliations:** 1 The Prince Charles Hospital, Brisbane, QLD Australia; 2 Faculty of Medicine, University of Queensland, Brisbane, QLD Australia; 3 Ipswich Hospital, Ipswich, QLD Australia; 4 St Andrew’s Ipswich Private Hospital, Ipswich, QLD Australia; 5 Rockhampton Base Hospital, Rockhampton, QLD Australia; 6 St Vincent’s Private Hospital Northside, Brisbane, QLD Australia; 7 Greenslopes Private Hospital, Brisbane, QLD Australia

**Keywords:** Colorectal neoplasms, Recurrence

## Abstract

**Background:**

Colorectal cancer (CRC) carries a significant disease burden worldwide. With the implementation of screening programs for colorectal cancer, the incidence of early-stage disease is increasing. As such, it is imperative to revise and optimise our risk stratification and treatment pathways for this patient cohort.

**Methods:**

This retrospective cohort study analyses clinicopathological features of early colorectal cancer from our institutional database between 2010 and 2020. Univariate and multivariable analyses were used to identify clinical and disease factors that may be associated with disease recurrence.

**Results:**

195 patients with stage one and two colorectal cancers were identified. Twenty-six patients experienced recurrence overall (13.3%). Recurrence rates were 8.4% and 17% in stage I and II disease, respectively. 19.2% of patients with recurrence did not possess any of the six commonly recognised risk factors. 5-year mortality rate for those patients who experienced recurrence was 61.5%. Using a multivariable model, venous invasion (*p* = 0.02), synchronous cancers (*p* = 0.017), microsatellite stability (*p* = 0.018) and peri-operative blood transfusion (*p* = 0.01) were found to be risk factors for recurrence.

**Conclusion:**

Early colorectal cancers carry a significant risk of recurrence and associated disease burden. Currently identified risk factors may not provide the full picture in terms of prognostic impact. Optimisation of risk stratification will assist to guide adjuvant therapy in this group of patients.

## Introduction

Colorectal cancer is a significant contributor to morbidity and mortality across the globe. It is the third most common cancer and the second leading cause of cancer-related death [1]. Over the past 20 years, advances in surgical technique, risk stratification and chemotherapeutic agents have been implemented with the aim of reducing recurrence and improving survival [2]. With many nations implementing population-based screening programs, the detection of colorectal cancer at its earlier stages is increasing [3]. However, even in early disease, recurrence after curative resection can occur. Therefore, appropriately prognosticating recurrence risk in early colorectal cancers is vital to tailor clinical decision making and treatment in this growing patient cohort.

Colorectal cancer recurrence is the result of microscopic residual disease, with lymph node involvement one of the most significant predictors [4, 5]. Adjuvant chemotherapy (AC) improves recurrence free survival (RFS) and overall survival (OS) in patients with lymph node involvement at diagnosis (AJCC stage III disease) and as such AC is the standard of care, unless precluded by medical comorbidities [4, 6, 7, 8]. For patients without lymph node involvement (stages I and II), the role of adjuvant therapy is less clear. Five-year recurrence rates in this patient group are between 10 and 25%, however the utility of AC on RFS and OS in stage I and II disease is controversial [5, 9, 10].

Therefore, efforts are being made to further stratify stage II disease into ‘low’ and ‘high’ risk of recurrence based on clinicopathological features. Commonly accepted risk factors include T4 disease, high grade tumours, clinical obstruction or perforation at presentation, presence of lymphovascular invasion or perineural invasion and inadequate lymph node sampling (< 12 nodes) [4, 7, 8]. The presence of each factor may increase risk of recurrence between 5 and 10%, with a cumulative effect for multiple factors [7, 10, 11]. As a result of this increased risk, AC for ‘high risk’ stage II patients is being considered [4, 7, 8]. As our understanding of colorectal cancer grows, more potential indicators of recurrence are discovered, with circulating tumour DNA (ctDNA), tumour budding, tumour-stroma ratio and micro-satellite stability being investigated [5, 7, 9].

Current international consensus does not recommend the use of AC in stage I disease irrespective of the presence of high risk features [4, 7, 8]. However, 5–18% of patients with stage I disease will also experience recurrence, and stage I disease can carry the same high-risk features as stage II disease [2, 12, 13, 14]. There is a paucity of data on risk stratification in stage I disease compared to stage II, however studies have found similar predisposition for recurrence when the same high-risk features are present [12, 13, 14].

As the incidence of early colorectal cancers rises, it is imperative to continue to investigate those patients who may be at higher risk of recurrence, and whether this risk can be mitigated with adjuvant therapy. This study aims to investigate commonly accepted ‘high risk’ features and more novel variables for their impact on recurrence risk in node-negative colorectal cancer. In doing so, we hope to contribute to the current efforts towards risk stratification of node-negative disease and identify potential target groups for novel adjuvant treatments and stringent follow up regimes.

## Methods

### Study design and population

This is a retrospective cohort study of patients diagnosed with stage I or II primary colorectal adenocarcinoma who underwent resection between January 2010 and December 2020 at our institution. Exclusion criteria were metachronous colorectal cancers, neoadjuvant chemotherapy and/or radiation and patients lost to follow up. Patient demographics and clinicopathological features (including histopathology, operative urgency, adjuvant therapy and follow up) were obtained from our institutions existing colorectal cancer database [15, 16]. Ethical approval was granted by the TPCH Human Research Ethics Committee (HREC/17/QPCH/295).

### Measures and definitions

AJCC guidelines (8th Edition) were used for the purposes of staging [17]. Emergency surgery was defined as surgery occurring withing 72 h of diagnosis. Locoregional recurrence was defined as relapse at the surgical site or in the lymph nodes directly draining this site. Any recurrence outside of this was deemed distant. The follow up period for this study was three years post curative resection. This timeframe was chosen as most colorectal cancer recurrences are expected to occur within this period [2]. Demographics and clinical data for patients lost to follow up were assessed to exclude bias.

### Statistical analysis

Variables were summarised using counts and percentages for categorical variables and median and interquartile range for continuous variables. Univariate analysis was run for all patient demographics and clinicopathological features to detect potential predictors of recurrence. T-test, Chi Squared and Fisher exact test were used as appropriate. Multivariable analysis including all variables was performed with logistic regression. Odds ratio with 95% confidence intervals were used. A p-value of < 0.05 was considered significant. Statistical analysis was performed using Stata v17.0 (STATA, College Station, Texas. USA).

## Results

277 patients underwent oncological colon resection for stage I and II disease. 82 of these patients did not reach three years of follow up, leaving 195 patients included in final data analysis. Median age at time of operation was 70 and the majority of patients were female (54%). 43% of patients had AJCC stage I colon cancer. (Table 1)

**Table 1:** Patient demographics and clinicopathological features– Included patients vs. lost to follow up

Variable	Included (%) *n* = 195	Lost to follow up (%) *n* = 82	*p*-value
**Age** ^†^	70 (59-77.5)	76.5 (70–83)	**< 0.001**
**Sex**			**0.007**
Male	89 (45.6%)	52 (63.4%)	
Female	106 (54.4%)	30 (36.6%)	
**BMI** ^†^	27.1 (24-32.4)	26.1 (23–30)	0.067
**ASA score** ^‡^			**< 0.001**
1	9 (4.7%)	1 (1.3%)	
2	75 (39.5%)	17 (22.1%)	
3	95 (50%)	45 (58.4%)	
4	11 (5.8%)	14 (18.2%)	
**Location**			0.905
Right	117 (60%)	51 (62.2%)	
Left	67 (34.4%)	26 (31.7%)	
Rectum	11 (5.6%)	5 (6.1%)	
**CEA** ^†^	2.8 (1.4–5.5)	2.8 (1.6–5.1)	0.326
**T Stage**			0.941
1	47 (24.1%)	22 (26.8%)	
2	36 (18.5%)	14 (17.1%)	
3	87 (44.6%)	37 (45.1%)	
4	25 (12.8%)	9 (11%)	
**Mucinous Component**			0.801
Absent	174 (89.2%)	74 (90.2%)	
Present	21 (10.8%)	8 (9.8%)	
**Grade**			0.808
Low	154 (80.2%)	66 (81.5%)	
High	38 (19.8%)	15 (18.5%)	
**Size (mm)** ^†^	37 (21–52)	34 (23-51.3)	0.859
**Synchronous**			0.242
No	191 (97.9%)	78 (95.1%)	
Yes	4 (2.1%)	4 (4.9%)	
**< 12 Nodes**			0.783
No	178 (91.3%)	74 (90.2%)	
Yes	17 (8.7%)	8 (9.8%)	
**Small Vessel Invasion**			0.529
Absent	174 (89.2%)	71 (86.6%)	
Present	21 (10.8%)	11 (13.4%)	
**Venous Invasion**			0.774
Absent	185 (94.9%)	77 (93.9%)	
Present	10 (5.1%)	5 (6.1%)	
**Tumour Infiltrating Lymphocytes**			0.753
Absent	142 (74.3%)	58 (72.5%)	
Present	49 (25.7%)	22 (27.5%)	
**Perineural Invasion**			0.366
Absent	170 (89%)	75 (92.6%)	
Present	21 (11%)	6 (7.4%)	
**Micro Satellite Stability**			0.398
MSS/MSI-L	112 (72.3%)	33 (66%)	
MSI-H	43 (27.7%)	17 (34%)	
**Transfusion**			0.089
No	174 (89.2%)	67 (81.7%)	
Yes	21 (10.8%)	15 (18.3%)	
**Emergency Surgery**			0.731
No	174 (89.2%)	72 (87.8%)	
Yes	21 (10.8%)	10 (12.2%)	
**Adjuvant Chemotherapy**			**0.010**
No	171 (87.7%)	80 (97.6%)	
Yes	24 (12.3%)	2 (2.4%)	

† Median and IQR, ‡ ASA: American Society of Anesthesiology score [18]; CEA: carcinoembryonic antigen; MSS: microsatellite stable; MSI-L: micro-satellite instability low; MSI-H: micro-satellite instability high

Note: not all patient records contained complete details for clinicopathological variables, and as such some data is missing.

Clinicopathological features were compared between those patients included and excluded (lost to follow up) from the study assess for statistically significant differences and identify any potential bias. (Table 1) There was significant variability in age (*p* < 0.001), sex (*p* = 0.007), ASA (*p* < 0.001), and adjuvant chemotherapy (*p* = 0.010).

26 (13.3%) of the 195 patients experienced a recurrence within the three year follow up period. (Table 2) Fifteen of these patients had distant recurrence (57.7%), nine had local recurrence (34.6%) and two had both (7.7%). Some patients experienced recurrence in multiple locations. (Table 3) Median time to recurrence was 367 days (range: 120–1091) and 5-year mortality rate for these patients was 61.5%.

**Table 2:** Recurrence by disease stage

	Total patients (%) *n* = 195	Stage I disease (%) *n* = 83	Stage II disease (%) *n* = 112
**No Recurrence**	169 (86.7%)	76 (91.6%)	93 (83%)
**Recurrence**	26 (13.3%)	7 (8.4%)	19 (17%)

**Table 3:** Distribution of sites of disease recurrence

Site of recurrence	Patients (%)
Liver only	9 (34.6%)
Lung only	5 (19.2%)
Liver and Lung	1 (3.9%)
Peritoneal/mesenteric	5 (19.2%)
Local and distal nodes	2 (7.7%)
Anastomosis	2 (7.7%)
Other	2 (7.7%)

In univariate analysis, CEA, T stage, tumour grade, venous invasion, perineural invasion, peri-operative transfusion, emergency surgery and adjuvant chemotherapy were found to have a significant impact on recurrence rate (Table 4). In multivariable analysis, higher rates of recurrence were associated with synchronous cancers (OR = 27.7; 95% CI 1.8-427.7), venous invasion (OR = 10.5; 95% CI 1.5–76.1), micro-satellite stable disease (OR = 26.6; 95% CI 1.8-402.2) and peri-operative transfusion (OR = 14.4; 95% CI 1.9-112.2).

**Table 4:** Comparison between patient groups based on recurrence status– Univariate and multivariable analysis

Variable	No Recurrence (%) *n* = 169	Recurrence (%) *n* = 26	*p*-value	Odds Ratio (95% CI)	*p*-value
**Age** ^†^	70 (59–77)	70.5 (55.5–80)	0.452	1.01 (0.96–1.07)	0.74
**Sex**			0.714		
Female	91 (85.8%)	15 (14.2%)		*REF*	
Male	78 (87.6%)	11 (12.4%)		0.49 (0.14–1.67)	0.26
**BMI** ^†^	27.1 (24.4–32)	27.8 (22.3–33.8)	0.528	1.07 (0.98–1.18)	0.11
**ASA** ^‡^			0.072		
1	9 (100%)	0 (0%)		*REF*	
2	69 (92%)	6 (8%)		735,946 (0-999999)	0.995
3	78 (82.1%)	17 (17.9%)		3,180,453 (0-99999)	0.995
4	8 (72.7%)	3 (27.3%)		6,601,988 (0-999999)	0.994
**Location**			0.774		
Left	59 (88.1%)	8 (11.9%)		REF	
Right	101 (86.3%)	16 (13.7%)		1.06 (0.26–4.26)	0.94
Rectum	9 (81.8%)	2 (18.2%)		2.14 (0.23–19.63)	0.50
**CEA** ^†^	2.6 (1.3–4.23)	7.4 (2.4–9.2)	**0.007**	1.01 (0.99–1.02)	0.37
**T Stage**			**0.036**		
1	44 (93.6%)	3 (6.4%)		REF	
2	32 (88.9%)	4 (11.1%)		1.30 (0.17–9.96)	0.80
3	76 (87.4%)	11 (12.6%)		1.20 (0.16–9.10)	0.86
4	17 (68%)	8 (32%)		3.56 (0.31–41.40)	0.31
**Mucinous Component**			0.168		
Absent	153 (87.9%)	21 (12.1%)		REF	
Present	16 (76.2%)	5 (23.8%)		2.77 (0.35–21.83)	0.33
**Tumour Grade**			**0.041**		
High	29 (76.3%)	9 (23.7%)		REF	
Low	137 (89%)	17 (11%)		0.41 (0.11–1.56)	0.19
**Size (mm)** ^†^	36 (21–51)	40 (26.3–53.8)	0.405	0.99 (0.97–1.03)	0.95
**Synchronous**			0.087		
No	167 (87.4%)	24 (12.6%)		REF	
Yes	2 (50%)	2 (50%)		27.70 (1.79-427.68)	**0.017**
**< 12 Nodes**			0.252		
No	156 (87.6%)	22 (12.4%)		REF	
Yes	13 (76.5%)	4 (23.5%)		3.10 (0.42–23.05)	0.27
**Small Vessel Invasion**			0.168		
Absent	153 (87.9%)	21 (12.1%)		REF	
Present	16 (76.2%)	5 (23.8%)		2.29 (0.35–14.90)	0.38
**Venous Invasion**			**0.005**		
Absent	164 (88.6%)	21 (11.4%)		REF	
Present	5 (50%)	5 (50%)		10.50 (1.50-76.05)	**0.02**
**Tumour Infiltrating Lymphocytes**			0.420		
Absent	121 (85.2%)	21 (14.8%)		REF	
Present	44 (89.8%)	5 (10.2%)		0.52 (0.11–2.54)	0.42
**Perineural Invasion**			**0.046**		
Absent	150 (88.2%)	20 (11.8%)		REF	
Present	15 (71.4%)	6 (28.6%)		1.09 (0.17-7.00)	0.93
**Micro Satellite Stability**			0.407		
MSI-H	39 (90.7%)	4 (9.3%)		REF	
MSS/MSI-L	96 (85.7%)	16 (14.3%)		26.56 (1.75-402.15)	**0.018**
**Perioperative Transfusion**			**0.041**		
No	154 (88.5%)	20 (11.5%)		REF	
Yes	15 (71.4%)	6 (28.6%)		14.40 (1.85-112.21)	**0.011**
**Emergency Surgery**			**0.011**		
No	155 (89.1%)	19 (10.9%)		REF	
Yes	14 (66.7%)	7 (33.3%)		1.83 (0.24–14.04)	0.56
**Adjuvant Chemotherapy**			**0.024**		
No	152 (88.9%)	19 (11.1%)		REF	
Yes	17 (70.8%)	7 (29.2%)		6.90 (0.60-78.81)	0.12

†Median and IQR ‡ ASA: American Society of Anesthesiology score [18]; CEA: carcinoembryonic antigen; MSS: microsatellite stable; MSI-L: micro-satellite instability low; MSI-H: micro-satellite instability high

Note: not all patient records contained complete details for clinicopathological variables, and as such some data is missing

## Discussion

This study investigated risk factors for recurrence in stage I/II colorectal cancer post curative resection. Recurrence was detected in 13.3% (8.4% and 17% in stage I and II disease respectively) of the population over the three-year follow up period. Recurrent disease was more often distant than locoregional (57.7% vs. 34.6%). The most common site of recurrence was the liver (34.6%) followed by the lung and peritoneum (both 19.2%). These results are comparable to current literature [4, 5]. Of the 26 patients who experienced recurrence, five (19.2%) did not display any of the six commonly accepted high-risk features [4, 7, 8]. This suggests further revision of the guidelines might improve detection of high-risk disease. Of those who experienced recurrence, five-year mortality was 61.5%.

Multiple potential risk factors for recurrence were revealed, including, pre-operative CEA, T stage, tumour grade, venous invasion, perineural invasion, peri-operative transfusion, emergency surgery and adjuvant chemotherapy. However, four variables, presence of synchronous cancers, venous invasion, microsatellite stable disease and perioperative transfusion were found to be independent risk factors for recurrence.

The term ‘lymphovascular invasion’ represents the presence of either venous or small vessel invasion, or both, as is recognised as a risk factor for disease recurrence. Small vessel invasion describes the presence of cancer cells in capillaries and lymphatics. In our study, the presence of venous invasion was independently associated with disease recurrence, however small vessel invasion was not a significant predictor of disease recurrence. Similar variation in prognostic significance between venous and small vessel invasion has been reported [19, 20]. Both venous invasion and small vessel invasion represent potential sources for residual microscopic disease and therefore theoretically both could result in disease recurrence. However, if clinically, they have different prognostic impacts it may be judicious to separate them in future classifications of high-risk disease.

Our study also revealed an independent association between synchronous colorectal cancers and recurrence. Currently, presence of synchronous cancers is not considered a risk factor for recurrence or indication for adjuvant chemotherapy [4, 21]. This is perhaps because there is currently no consensus as to the impact of synchronous cancers on prognosis and recurrence [21, 22, 23]. This population is difficult to study in terms of recurrence risk given their low prevalence, comprising only 3.5% of colorectal cancers and their association with known higher risk groups such as in Lynch Syndrome, which has its own prognostic impacts [22]. Our study is limited in that only a small number of patients had synchronous cancers, and therefore our results need to be interpreted with caution. However, our findings are in keeping with studies identifying an increased risk of cancer recurrence in the setting of synchronous cancers [24, 25]. Synchronous disease as a potential risk factor for recurrence therefore warrants further investigation.

This study also found that micro-satellite stable tumours or those with low frequency instability were associated with recurrence. There are distinct molecular and clinicopathological differences between microsatellite stable and instable disease and their response to adjuvant therapies [26]. MSI-H disease is associated with disease factors of both positive and negative prognostic significance, however, there is a general suggestion that dMMR may have an overall prognostic benefit in colorectal cancer [26, 27]. Other studies have also shown similar results with improved disease-free survival in dMMR disease, and that dMMR nearly halves the risk of disease recurrence [10, 27]. Unfortunately, as our study began in 2010, many of the earlier cases did not include MMR testing. However, our results are in keeping with previously published literature.

Peri-operative blood transfusion was also associated with disease recurrence in multivariable analysis. Autologous blood transfusion can increase risk of short term post-operative complications, however its impact on disease recurrence is an area of ongoing contention [28, 29, 30]. It is theorised that allogenic transfusion in the post-operative period stimulates the production of inflammatory and immunosuppressive mediators which impair the body’s ability to fight microscopic residual disease [29]. At present, peri-operative transfusion is not a considered variable in the decision-making pathway for adjuvant therapy, however the current data warrants further investigation into its role in recurrence.

This study has several limitations. Firstly, this is a single-centre study which reduces external validity, as surgical technique plays a large part in colorectal cancer outcomes. Our study utilised a follow up period of three years, as the majority of recurrences occur within this timeframe, however this may have excluded some cases of recurrence, particularly as time to recurrence increases with lower stage disease [2, 31]. There were also a considerable number of cases lost to follow up for primarily undocumented reasons. To detect potential bias arising from the exclusion of these patients, comparison of clinicopathological features between those included and excluded from the study was performed. This revealed that age, sex, ASA and adjuvant chemotherapy status were the only variables that differed significantly between the groups. Importantly, none of these variables were found to be statistically significant risk factors for recurrence in the final multivariate analysis. Due to our study’s small sample size, we chose not to perform sub-group analysis with stage I and II disease to preserve statistical power. We utilised multivariable analysis including T stage as a variable to account for potential confounding between the two stages of disease. Contents of histopathological reporting also changed throughout the study period, and there was a significant proportion of data missing for certain variables.

This study also does not include several other variables that have been suggested as predictors of disease recurrence, including ctDNA and tumour budding [7]. The DYNAMIC trial has recently published results to suggest that using ctDNA levels to guide the use of AC results in fewer patients requiring AC without any significant difference in recurrence [32]. At present, ctDNA and tumour budding are not routine investigations at our institution, but we acknowledge their potential for risk prognostication.

Overall, this study contributes to the literature citing a link between venous invasion and recurrence of disease. It also proposes synchronous disease, perioperative transfusion and microsatellite stable disease as areas for further investigation. Defining the patients that are at high risk of recurrence provides a target for further research into mitigating these risks, either in the form of novel adjuvant therapies or rigorous follow-up surveillance, to continue to reduce disease burden.

## Data Availability

The datasets used and analysed during the current study are available from the corresponding author on reasonable request.
